# Contributing Role of High Mobility Group Box 1 Signaling in Oral Cancer Development and Therapy

**DOI:** 10.3390/life13071577

**Published:** 2023-07-18

**Authors:** Grigorios Plemmenos, Valentini Tzimogianni, Christina Fili, Christina Piperi

**Affiliations:** 1 School of Dentistry, National and Kapodistrian University of Athens, 2 Thivon Str, Goudi, 11527 Athens, Greece; plemmenosgreg@windowslive.com; 2 Department of Biology, Medical School, National and Kapodistrian University of Athens, 75 M. Asias Street, 11527 Athens, Greece; vtzimogianni@gmail.com; 3 Medicine and Surgery, Department of Pharmacy and Medicine, Sapienza Universita di Roma, Piazzale Aldo Moro 5, 00185 Roma, Italy; 4 Department of Biological Chemistry, Medical School, National and Kapodistrian University of Athens, 75 M. Asias Street, 11527 Athens, Greece

**Keywords:** oral cancer, therapy, HMGB1, RAGE, prognosis

## Abstract

Oral squamous cell carcinoma (OSCC) is the most frequent type of oral cancer of multifactorial origin, characterized by histological and clinical manifestations. To date, there are no specific biomarkers or treatment modalities available to efficiently manage this neoplasia, demanding further research on the molecular background of OSCC pathology. Elucidation of signal transduction pathways and associated molecules with differential expression and function in OSCC are expected to enhance the future development of molecular targeted therapies. Among signaling proteins with a potential functional role in OSCC, the High Mobility Group Box 1 (HMGB1) protein has stimulated scientific interest due to frequent upregulation, and implication in the progression of many types of head and neck cancer types. HMGB1 is a nuclear nonhistone protein and an extracellularly secreted cytokine that can interact with several signaling molecules implicated in the pathogenic pathways of OSCC. Binding of HMGB1 to specific receptors on OSCC cells such as the receptor of AGE (RAGE) and the toll-like receptor (TLR) has been shown to initiate several intercellular signaling cascades that can promote OSCC growth, invasion, and metastasis, indicating a potential target for patient prognosis and therapeutic approaches. The purpose of this review is to explore the functional role and associated signaling of HMGB1 in OSCC in order to reveal potential therapeutic targeting options.

## 1. Introduction

The highest incidence rate of Head and Neck Squamous Cell Carcinomas (HNSCCs) involves the oral cavity with Oral squamous cell carcinoma (OSCC) representing the majority of HNSCC cases [1]. According to the Global Cancer Observatory (GCO) estimates, OSCC prevalence in 2020 was over 377,713 cases, referring only to cancer types arising at the oral cavity [2]. OSCC presents one of the most prevalent cancer types in Asia, while it is rather rare in Western countries [3]. The rapid increase in oral cancer cases seems to take place after the age of 50 years, particularly during the seventh decade, affecting mostly Hispanic and Black males [4]. OSCC morbidity and mortality rates remain the greatest among HNSCCs patients while the main etiological factors include tobacco smoking, alcohol consumption and chewing, which can alter intracellular signaling and the oral cavity’s environment [5]. Current OSCC treatment protocols include surgery, radiation, and chemotherapy in various combinations based on disease presentation and pathological characteristics [5]. However, oral mucositis characterized by edema, irritation, inflammation, and ulcerations of oral mucosa presents a common debilitating side effect for patients undergoing chemotherapy or radiotherapy [6].

OSCC commonly presents as dysplastic lesion in the epithelium characterized by irregular stratification and loss of apical-basal polarity of epithelial cells, with enlarged nuclei, loss of intercellular adhesion and abnormal polarization. Oral leukoplakia (OLK) is the most common form and proliferative verrucous leukoplakia (PVL) is the most widespread, multifocal, and aggressive type [5,6].

The underlying molecular mechanisms in the development of OSCC involve the dysregulation of the cell cycle cyclin/cyclin-dependent kinase (CDK) complex which phosphorylates Retinoblastoma (RB) tumor suppressor protein at the level of G1/S transition [5,6]. Additionally, overexpression of transforming growth factor-alpha (TGF-α) presents an early marker for OSCC development, playing a significant role in the hyperplasia and invasiveness associated with its progression [6]. From a genetic perspective, oral cancer has been shown to involve alterations in *Ras* oncogene and anti-apoptotic B-cell lymphoma 2 (Bcl-2) family proteins as well as dysfunction of the *TP53* tumor suppressor gene [5,6]. The main intracellular signaling pathways involved in proliferation of OSCC cells include the Ras/extracellular signal-regulated kinases (ERK)/mitogen-activated protein kinases (MAPK) axis and the phosphatidylinositol 3-kinase (PI3K)/Akt/mammalian target of rapamycin (mTOR) pathway, which have an additional impact on apoptosis, migration, and cell metabolism [6]. To this end, a constant search of molecular characteristics and cell signaling pathways associated with the disease, has revealed the implication of the High Mobility Group Box 1 (HMGB1) protein in carcinogenesis, exhibiting a dual functional role, both oncogenic and tumor suppressive depending on cancer type and context, as well as a biomarker potential for diagnosis and therapy [7]. 

HMGB1 belongs to the large family of HMG proteins exhibiting high electrophoretic mobility along with HMGB2. HMG proteins constitute the second largest group of nuclear proteins apart of histones and they are structurally distinguished into three families: High Mobility Group A (HMGA), High Mobility Group B domain proteins (HMGB1-4), and High Mobility Group N (HMGI/Y) [8]. All family members possess an HMGB domain (~80 aa) which is a characteristic structural and functional motif [8]. HMGB1 and HMGB2 bind to DNA without following any sequence specificity, acting mainly as transcription factors [7,8,9]. Both HMGB1 and HMGB2 proteins present similarities in amino acid sequence and structure, consisting of two DNA-binding domains A and B, a short N-terminal region, and a C-terminal sequence rich in aspartic and glutamic acids [10,11]. HMGB1 differs from HMGB2 at the C-terminal length consisting of 30 and 20 amino acids and being involved in spatial rearrangement of A and B domains as well as in the regulation of proteins interaction with nucleic acids or other proteins. HMGB1 is the best studied member of the family exhibiting a variety of functions and being implicated in several immune, metabolic, and inflammatory diseases, including cancer [12,13]. 

HMGB1 function is ultimately linked to its localization in the intercellular space. Extracellular signals, redox status, and post translational modifications trigger HMGB1 release from the nucleus, induce its translocation to the cytoplasm, and subsequently to the intercellular space, where it can induce signaling in response to cell damage and necrosis or trigger the immune response. HMGB1 contains two nuclear localization sequences (NLS) with four and five lysines each and two non-classical nuclear export sequences (NES). These two sequences are responsible for the protein shuttling between the nucleus and the cytoplasm. There is evidence that post-translational modifications of NLS or NES can regulate the accumulation of proteins either in the nucleus or in the cytoplasm [14,15]. Acetylation of the lysines of the two NLS leads to HMGB1 hyperacetylation and consequently its translocation to the cytoplasm. In the opposite case, HMGB1 remains in the nucleus. Activation of the JAK/STAT1 pathway has been shown to mediate the acetylation of HMGB1. During inflammation, both LPS and interferon have been shown to bind to TLR4, activate the JAK/STAT1 pathway and induce HMGB1 acetylation [14]. On the other hand, lysine deacetylation inhibits the cytoplasmic translocation of HMGB1, and treatment with histone deacetylase inhibitors was shown to promote acetylation and translocation of HMGB1 to the cytoplasm, affecting cell proliferation [15].

Other post-translational modifications such as phosphorylation and methylation can also regulate HMGB1 localization. Phosphorylation reduces the binding ability of HMGB1 to KAP-aα1, a nuclear import protein and promotes HMGB1 localization. In addition, HMGB1 has been shown to trigger inflammatory responses through its intramolecular disulfide bond formation, which also involves a post-translational modification [14]. Lastly, HMGB1 contains three cysteines whose oxidation causes reduced inflammatory activity both in vitro and in vivo [15].

Additionally, HMGB1 can be secreted extracellularly from pro-inflammatory cells as well as cells undergoing stress, or necrosis during inflammation. HMGB1 activation has been associated with cytokine activity, involved in macrophage secretion of pro-inflammatory cytokines [16], mediated by the B domain and antagonized by the A domain [17]. HMGB1 has been also involved in both innate and adaptive immunity as well as in many biological processes including transcription and DNA repair, cell signaling, migration, and associated pathogenic mechanisms.

Emerging research evidence indicates that HMGB1 can exhibit an extra- and intracellular role in cancer development, progression, and therapy [12]. Extracellular HMGB1 has been shown to participate in tumor formation and metastases, acting as a pro-tumor factor, whereas intracellular HMGB1 exhibits pro-autophagic activity, promoting cancer progression and increasing drug resistance [11]. The major signal transduction axis of HMGB1 in cancer seems to be mediated through the receptor for advanced glycation end products (RAGE) and toll-like receptor (TLR)-4 [12]. Activation of the HMGB1-RAGE signaling axis activates MAPK pathways, inducing Nuclear Factor-κB (NF-κB) activity that is ultimately linked to inflammatory response and cancer cell proliferation, as well as tumor invasiveness [18]. Knockout of RAGE has been shown to decrease tumor progression and metastasis while increasing resistance to chemotherapy [19], thus indicating a potential target of therapeutic intervention. 

The present review explores the involvement and functional role of HMGB1 in OSCC pathogenesis, focusing on the associated signal transduction mechanisms and current targeting options in order to reveal potential therapeutic regimens or interventions.

## 2. Evidence of HMGB1 Involvement in OSCC Pathogenesis

A research study investigating different HMGB1 genetic polymorphisms in OSCC and oral lichen planus patients, has shed light on OSCC risk, progression, and prognosis [9]. The HMGB1 1177G/C genotype was detected in OSCC and suggested as a potential marker of tumor stage and progression. However, this Single Nucleotide Protein (SNP) was shown to be a negative prognostic marker only in recurrence-free patients and not in the overall survival rates. Moreover, HMGB1 gene haplotypes (AGC and GGC) were shown to be correlated with elevated risk of oral cancer and oral lichen planus progression to OSCC [9]. Furthermore, another study investigating HMGB1 genetic variations among patients, detected four SNPs that were associated with OSCC. Among them, the rs1045411 shares a binding site with hsa-miR-505-5pmiRNA which increases DNA instability and susceptibility to OSCC. It was also shown that environmental factors like cigarette smoking, and betel chewing elevated the incidence of HMGB1 SNPs, enforcing its connection to OSCC onset. Another study evaluating the prevalence of every HMGB1 SNP with OSCC risk, revealed that rs1412125 and rs2249825 are involved in HMGB1 transcriptional regulation, further suggesting HMGB1 as an independent predictor of OSCC progression and prognosis [20]. Of interest, there was no association detected between HMGB1 levels in the primary lesion and late neck metastasis in a cohort of 26 patients diagnosed with early tongue OSCC. During the 7-year follow-up, 10 patients were detected with neck metastasis without, however, any signs of local recurrence or distant metastasis. Moreover, no significant difference was found in HMGB1 expression between the two groups, indicating that HMGB1 cannot be used as a predictive marker for metastasis [12].

Additional evidence of HMGB1 involvement in OSCC pathogenesis comes from studies detecting increased levels of the HMGB1 ligand, receptor for AGE (RAGE) with prognostic significance in OSCC [21,22]. Upregulation of RAGE expression has been observed in OSCC compared to normal oral mucosal and salivary glands which exhibit no RAGE expression. Moreover, high expression of RAGE in OSCC was significantly correlated with the depth of invasion and OSCC recurrence [21]. Although RAGE immunoreactivity was not further associated with the clinical stage or other histopathological parameters, a connection between disease-free survival rate and RAGE expression was detected in OSCC patients by multivariate analysis [21]. The low expression of RAGE was correlated with better prognosis, proposing RAGE as an independent prognostic marker for OSCC patients’ post-treatment, who are vulnerable to cancer recurrence and should register to a follow-up care schedule [21]. A more recent study in Iranian patients with OSCC showed that HMGB1 detection in the blood as well as in the neoplastic tissues of OSCC patients was highly correlated with tumor size and lymph node involvement [22]. 

## 3. Molecular Mechanisms of HMGB1 Implication in OSCC Pathology

There are several studies demonstrating the highly significant role of HMGB1 in HNSCC and OSCC. HMGB1 has been implicated in the proliferation and migration of cancer cells, tumor-escape, and metastasis. HMGB1 activity has been associated with RAGE independent and RAGE dependent pathways. On RAGE independent pathways, HMGB1 interacts with erythropoietin-producing human hepatocellular B4 (EPHB4), NF-κB, TLR4 and melanoma inhibitory activity (MIA) protein to induce its tumorigenic effects and affect autophagy. On the other hand, HMGB1-RAGE signaling pathways have been highly associated with pain signals, Epithelial-to-Mesenchymal Transition (EMT) process, metastasis, bone invasiveness and angiogenesis in OSCC.

### 3.1. RAGE Independent Mechanisms

Studies on cell proliferation and metastasis have revealed that the erythropoietin-producing human hepatocellular B4 (EPHB4) is an important regulator of tumor functions in OSCC, associated with lymph node metastasis, differentiation, and poor prognosis. EPHB4 belongs to the receptor tyrosine kinases (RTK) family, with widespread roles in physiological and pathological processes, and frequently upregulated in cancer cells through activation of the PI3K signaling pathway and related to poor prognosis [23].

Downregulation of EPHB4 was shown to induce destabilization of HMGB1 protein and downregulation of NF-κB pathway molecules (phospho (p)-p65, phospho-nuclear factor of kappa light polypeptide gene enhancer in B-cells inhibitor alpha, p-IκBα) in OSCC cells. Additionally, targeted knockdown of HMGB1 expression induced a reduction in p-p65, while EPHB4 levels remained unchanged. These data imply that NF-κΒ pathway functions as a downstream effector pathway of HMGB1 protein in OSCC cells and as a potential regulator of cancer cell proliferation and migration [24].

Major compounds of the OSCC tumor microenvironment are Tumor-Associated Macrophages (TAMs). These specific types of macrophages encompass mostly M2 type macrophages with tumor promoting properties such as immuno-suppression, neovascularization, angiogenesis and stromal activation and remodeling, while M1 type (proinflammatory classically activated macrophages) can also be seen, leading to the transformation of OSCC cells to cancer stem cells. HMGB1, that is either produced by OSCC cells or macrophages, can induce TLR4 expression, NF-κB/p65 activation and increased Interleukin 10 (IL-10)/transforming growth factor beta (TGF-β) expression which all lead to TAMs polarization. In OSCC cells, HMGB1 was shown to be involved in macrophage recruitment via the IL-6/(signal transducer and activator of transcription 3) STAT-3 pathway. On the other hand, HMGB1 produced by the tumor-specific macrophages can regulate the microenvironment through IL-6/STAT3/(programmed death-ligand 1) PD-L1 pathway and IL-6/NF-κB/(matrix metalloproteinase 9) MMP-9 pathway leading to further immunosuppression and aggressive phenotypes of OSCC cells. The polarization of macrophages, along with the additional release of cytokines and the general formation of an immunosuppressive tumor microenvironment, indicates HMGB1 protein as an important target for OSCC immunotherapy [25].

Moreover, HMGB1 is commonly expressed on inflammatory, stressed, and necrotic cells as a Danger Associated Molecular Pattern (DAMP) protein and increases substantially in cancer cells as a tumor-derived danger signal [26]. T regulatory cells (Tregs) possess receptors for HMGB1, such as TLR4 and RAGE, which are also expressed on cells of innate immunity. HMGB1 is a highly chemoattractant molecule, leading Tregs to the tumor site and suppressing their T-cell proliferative activity, thus promoting a tumor-escape environment in HNSCC patients [27], including OSCC patients. Additionally, HMGB1, as a potent innate immune mediator, has been shown to be involved in the onset of oral mucositis after chemotherapy and radiotherapy treatment [26]. DNA damage and elevated oxidative stress in basal epithelial cells lead to mitochondrial dysfunction which induces translocation of HMGB1 from the nucleus to the cytoplasm and its extracellular release. HMGB1 has been suggested as an independent predictor of the severity of oral mucositis as well as a possible target for treatment [26].

HMGB1-overexpressing cancer cells concomitantly express the pro-metastatic Melanoma Inhibitory Activity (MIA) protein [10]. MIA is a secretory protein, inextricably linked with invasion and metastasis in melanoma cells and its expression is enhanced by HMGB1 through interaction with NF-κB p65. Regarding MIA and HMGB1 in OSCC, it has been shown that HMGB1 and NF-κB p65 were considerably higher in the metastatic OSCC line, HSC3 than in the non-metastatic OSCC cell line, HSC4. More importantly, they also showed that HMGB1–NF-κB p65 interaction was significantly increased in HSC3 in comparison to HSC4. In addition, HMGB1 antisense treatment was shown to decrease MIA expression in the metastatic human OSCC cell line, further indicating that the interaction of intracellular HMGB1 and NF-κB p65 enhances MIA expression [10].

Another cellular process that HMGB1 regulates in OSCC is autophagy which functions to degrade damaged or aged proteins, using them as biofuels in eukaryotic cells. Autophagy serves as a recycling program to maintain ATP production and cell homeostasis. Studies have revealed that autophagy keeps cancer stem cells viable, promotes EMT signaling and thus, OSCC [28]. However, there is also contradictory evidence indicating that autophagy may also inhibit apoptosis, migration, and invasion of OSCC cells. These contradictory data have been attributed to the different stages of OSCC used in different studies [28]. HMGB1 has been shown to increase pro-survival autophagy through binding to Beclin-1, a key regulator of autophagy [7] and inhibit apoptosis, in several clinical settings, including OSCC. Exposure of OSCC cells to hypoxia was further shown to induce autophagy by activating the signaling pathway of Hypoxia-inducible factor 1-alpha (HIF-1α)/Bcl-2 interacting protein 3 (BNIP3)/Beclin-1 and inducing HMGB1 secretion, thus acting as a survival mechanism for the cancer cells [29].

### 3.2. RAGE Mediated Mechanisms

RAGE is a pattern recognition receptor of the cell surface immunoglobulin (Ig) superfamily proteins. Its extracellular domain (the ectodomain) comprises of three Ig domains (V, C1, C2) with the V domain being responsible for ligand binding, followed by the transmembrane and cytoplasmic domains. RAGE is a multiligand receptor that can sense endogenous stress signals. Among the ligands that can bind to its extracellular part are HMGB1, Advanced Glycation End products (AGEs), amyloid β oligomers, S100 proteins, glycosaminoglycans, DNA and complement components. RAGE signaling and its ligand interactions have been implicated in innate immunity, chronic inflammatory processes, and cancer progression [30].

RAGE is detected at low levels in normal tissues and becomes upregulated upon increased concentration of its ligands, like HMGB1. Studies have shown that RAGE and HMGB1 silencing suppressed the generation and metastatic potential of HSC4 cells [31], further indicating that RAGE expression is involved in OSCC pathogenesis.

Apart from RAGE interaction, extracellular HMGB1 can also bind to TLR4 inducing neuronal signaling and being implicated in neuropathic pain. Western blot analysis of mouse and human HNC cell lines showed that bone and neuronal cells have RAGE and TLR4 receptors and thus express high levels of HMGB1 which contribute to neurite sprouting. To evaluate the effects of HMGB1 in the Dorsal Root Ganglia (DRG) sensory cells, injection of HNC cells, SAS-CM with and without HMGB1 antibody showed that hyperalgesia and increased neuron excitation may be attributed to HMGB1 administration to the specific areas. Moreover, it was shown that HMGB1 has three different redox forms (expressing Cys23, Cys45 and Cys106), two of which were shown to stimulate the DRG neuron cells by increasing p-ERK [32].

**Figure 1:**
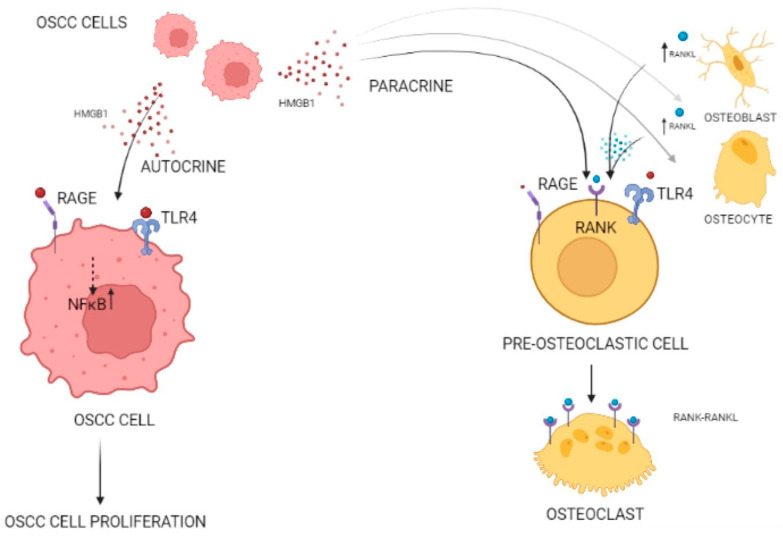
The dual role of HMGB1 expression in OSCC proliferation and bone invasion. OSCC cells express HMGB1 in an autocrine and paracrine manner. On the left side of the figure, HMGB1 produced by an OSCC cell is recognized by nearby OSCC cells through RAGE and TLR4 receptors, leading to the upregulation of the NF-κB pathway in their cell nuclei and promotion of their cell proliferation (autocrine manner). On the right side, HMGB1 elicited by an OSCC cell is recognized by osteoblasts, osteocytes, and pre-osteoclastic cells. At the former two cell types, recognition of HMGB1 leads to the upregulation of RANKL secretion. Regarding pre-osteoclastic cells, HMGB1 is recognized by RAGE and TLR4 receptors on the cell membrane. RANKL secreted by osteoblasts and osteocytes is recognized by RANK on pre-osteoclastic cells. In turn, the maturation of the pre-osteoclastic cells into osteoclasts induces bone invasion (created with BioRender, accessed on February 2023). TLR4, toll-like receptor 4; RAGE, receptor for AGE; NF-κB, nuclear factor-κB, RANK, receptor activator of nuclear factor-κB; RANKL, RANK-Ligand.

Moreover, HMGB1 is also overexpressed in bone invasive OSCC cells and activates the RAGE signaling pathway. Among several OSCC cell lines tested, the highly malignant and metastatic SAS cells showed the greatest overexpression of HMGB1. In this cell line, administration of HMGB1 neutralizing antibody and a RAGE-specific inhibitor suppressed OSCC cell proliferation. Meanwhile, HMGB1 neutralizing antibody efficiently attenuated the proliferation of pre-osteoclastic cells RAW.264.7, suggesting the dual role of HMGB1 in the invasion of cancer cells into the bone. It is thus evident that HMGB1 promotes OSCC cell proliferation autocrinally, while in a paracrine manner enhances pre-osteoclast maturation (Figure 1).

HMGB1 exerts its actions on bone metabolism by activating RAGE and TLR4 pathways in osteoclasts while upregulating RANKL expression in osteoblasts and osteocytes [33].

Furthermore, HMGB1 has been shown to promote EMT signaling in several cancer types via the RAGE-NF-κB signaling pathway [7]. The EMT process involves loss of epithelial features and induction of a mesenchymal phenotype, by activation of related genes. EMT process alters the migratory properties of cancer cells and enables their invasion to surrounding tissues [7,10]. EMT signaling has also been shown to take place in OSCC cells invasive behavior and contribute to metastasis [10]. Several miRNAs and long non-coding RNAs have been shown to induce EMT phenotype in oral cancer by regulating different transcription factors and signaling pathways [34]. Among them miR-155-5p expression was negatively correlated to E-cadherin and was shown to enable EMT signaling by TGF-β1 and PI3K/glycogen synthase kinase 3 (GSK-3)/β-catenin pathway, contributing to OSCC progression and predicting relapse of patients with early-stage OSCC. Additionally, miR-29b-1-5p was shown to also target E-cadherin gene (*CDH1*) and work along with the *c-Met* oncogene to induce EMT signaling in OSCC while miR-199a-5p overexpression blocked EMT signaling and inhibited migration and cell invasion [34]. Several lncRNAs, including the lncRNA MALAT1 have been shown to modulate the activity of HMGB1 as well as its upstream receptors, affecting EMT signaling and presenting useful therapeutic targets [35]. 

More data advocate for EMT signaling involvement in OSCC pathogenesis. After the confirmation of elevated HMGB1 mRNA levels in cancer cells (SCC-4, SCC-9, SCC-15, TCA-811 and CAL-27) in comparison with the neighboring non-cancerous tissues, further investigation was conducted on SCC-9 cell line as the one with the highest expression of HMGB1. siRNA HMGB1 effectively downregulated HMGB1 expression in SCC-9 cells, though resulting in reduced cancer cell colony formation, as well as in increased cell apoptosis providing clear evidence on the influence of HMGB1 on the proliferation and viability of the tumor cells. Analogous findings were obtained by the examination of the invasion potential of the cancer cells after HMGB1 silencing. Regarding EMT pathway, these series of experiments demonstrated that HMGB1 regulates the establishment of the EMT process in OSCC from the nucleus, where it is accumulated, and not from the cytoplasm. On the one hand, nucleic HMGB1 is responsible for E-cadherin suppression and elevation of the mesenchymal transcription factors Snail, Slug as well as N-cadherin expression, thus being implicated in OSCC proliferation and invasion [36]. 

In addition, RAGE has been implicated in the process of angiogenesis which contributes to the progression of OSCC. The angiogenic factors Vascular Endothelial Growth Factor (VEGF), IL-8, basic fibroblast growth factor (bFGF), and platelet-derived endothelial growth factor (PDGF) have been detected in OSCC and were correlated with tumor microvessel density (TMD), a representative marker of angiogenesis [37].

When VEGF and VEGF-C were investigated as major ligands of RAGE upon human recombinant HMGB1 administration in OSCC cell lines, it was shown that VEGF secretion was significantly upregulated in RAGE-positive tumor cells in a dose-dependent manner. However, VEGF-C levels were not significantly altered suggesting that RAGE does not induce lymphangiogenesis in OSCC [38]. Therefore, it was suggested that HMGB1-RAGE may be involved in tumor growth, but further investigation is needed since RAGE expression depends on histological tumor type and intracellular signaling.

## 4. Targeting Potential of HMGB1 in OSCC

It is evident that HMGB1 is ultimately involved in the development and progression of cancer as well as in angiogenesis and metastasis, pointing towards HMGB1 targeting as a potential therapeutic approach. To this end, HMGB1 inhibitors have been developed and previously tested as therapeutic agents in several cancer types, including breast and non-small lung cancer. HMGB-A box, a competitive antagonist of HMGB1, and glycyrrhizin, a small molecule inhibitor as well as RAGE antagonist peptide and ethyl pyruvate have been shown to block extracellular HMGB1 and affect cell proliferation and invasion in vitro, as well as leading to tumor growth suppression in vivo [38,39]. Currently, a Phase 1 multicenter clinical trial is in progress, evaluating the pharmacodynamics, safety, and anti-tumor activity of the orally available small molecule HMGB1 inhibitor SB17170, in patients with metastatic solid tumors who have failed previous treatment (SB17170 clinicaltrials.gov). SB17170 was previously shown to exert anti-tumor effects in B16F10 murine syngeneic models. However, no effects were observed in immunodeficient mice indicating that the adaptive immune response is required for the anti-tumor effects of HMGB1 [40]. 

Regarding OSCC, surgery and radiotherapy are selected as therapeutic regimes for stages 1 and 2 while chemotherapy is administered as part of combined treatment to improve local tumor control and survival rates for stages 3 and 4 [2,3,31]. Molecular therapies are highly demanded to improve current therapeutic schemes in OSCC and HMGB1 signaling has been therefore explored by several studies as a possible targeting approach (Table 1). Oral mucositis is often a cancer treatment side-effect, characterized by swelling, erythema, pain, inflammation, and ulceration, presenting a rather debilitating complication [41]. HMGB1 as a known DAMP has been detected as a potential contributing factor in the pathogenesis of oral mucositis associated with the underlying oxidative stress and inflammation. A study using an HMGB1 inhibitor, the tetrahydropyran-4-yl)-[2-phenyl-5-(1,1-dioxo-thiomorpholin-4-yl) methyl-1Hindole-7-yl] amine (NecroX-7), which exhibits free radical scavenging properties, was shown to block the release of HMGB1 in oral mucosa in chemotherapy-induced animal models. It was demonstrated that NecroX-7 exhibits a significant efficacy in decreasing the incidence of ulceration and the severity of oral mucositis in a dose-dependent manner [26]. Furthermore, NecroX-7 suppressed reactive oxygen species (ROS) levels and the DNA damage response marker γH2AX that is usually increased upon mitochondrial-induced apoptosis due to chemotherapy [26]. The administration of NecroX-7 further abolished p53 phosphorylation and decreased PUMA and Bax expression as it was shown in Western Blot and PCR, restoring Bax/Bcl2 ratio as well as caspase-3 to control levels. Also, real time PCR analysis showed that NecroX-7 can block the NF-κB pathway and the excessive production of inflammatory cytokines, decreasing further the inflammatory reactions of radiation and chemotherapy [26]. Moreover, NecroX-7 was tested for its anti-tumor activity in combination with other therapeutic modalities in mice but showed no significant modulation of 5 Fluorouracil (5FU) anti-tumor activity or radiation [26] (Table 1). 

Alteration of host immune responses against cancer cells is a novel research field, where specific molecules are used to re-activate the repressed ones by cancer immune system. One of them, JQ1, is a potent and very specific small transmembrane inhibitor for the bromodomain and extra-terminal (BET) protein family [42]. Studies have shown that JQ1 can affect the expression of other family members of BET proteins and that it can suppress cancer cell proliferation through regulation of gene expression in many types of cancer. In vitro experiments in two different cell lines for OSCC, CAL-27 and SCC7, indicated that JQ1 caused HMGB1 release to the extracellular environment without, however, increasing intracellular HMGB1 levels, as expected [42]. Therefore, it was assumed that JQ1 may elicit HMGB1 release but not its expression. Consequently, extracellular HMGB1 along with JQ1-induced release of calreticulin (CALR), and ATP will provoke immunogenic apoptosis of tumor cells. The following in vivo experiment ascertained the anti-tumor effects of JQ1, since mice treated with JQ1 presented a higher T-cell infiltration and smaller tumor dimensions. Thus, JQ1 induces cell death eliciting an immune response through HMGB1 release and could be a possible target for therapeutic approaches of OSCC [42].

The release of HMGB1 in a tumor microenvironment can be also elicited both by hyperthermia and hypoxia, but hyperthermia was shown to exert a stronger effect [29]. HMGB1 secretion as well as apoptosis rate were elevated in OSCC cells upon exposure to high-temperature and chemotherapy [26]. Moreover, the combined administration of hyperthermia and chemotherapy resulted in significantly greater induction of apoptosis and HMGB1 release [29]. The combined use of autophagy inhibitors 3-Methyladenine (3-MA) and YC-1 (an inhibitor of HIF-1α), attenuated HMGB1 release in cancer cells, indicating a potential underlying mechanism regulating extracellular levels of HMGB1 and autophagy in OSCC cells [29]. Indeed, autophagy has been correlated with the radio resistance displayed by cancer cells [43]. More specifically, a remarkable increase in autophagy was observed in cancer cells irradiated with a dose of 60G in comparison with non-irradiated cancer cells. However, no difference was observed between radiated and non-radiated cells in terms of total amount of HMGB1. Another study showed the translocation of HMGB1 from the nucleus where it is normally detected, to the cytoplasm and at the extracellular area of radioresistant cells. The cytoplasmic HMGB1 was shown to be bound with Beclin-1, suggesting a synergistic effect in autophagy. However, metastatic cancer cells in lymph nodes did not exhibit analogous alterations in autophagy flux [26]. 

Additionally, HMGB1 regulation of autophagy has been studied in OSCC cells treated with the chemotherapeutic drug vincristine. Vincristine exhibits anti-cancer effects by enhancing cell apoptosis, while HMGB1 exhibits the opposite role by upregulating the anti-apoptotic myeloid leukemia 1 (MCL-1) protein levels, thus contributing to the autophagy-mediated protection of cancer cells [44]. Regarding the involvement of HMGB1 in immune responses, human leukemia monocytic cell line THP-1-derived macrophages exhibited significantly increased IL-6 levels in OSCC CAL-27 cells exposed to the immunostimulant polyinosinic:polycytidylic acid (poly(I:C) for 24h and after exposure to palliative 8Gy/single fraction radiation. Consistent with this finding, HMGB1 neutralizing antibody was used to inhibit macrophage recruitment promoted by poly(I:C) and radiation in CAL-27 conditioned cells. Thus, HMGB1 may serve as a possible target for inhibiting recruitment of macrophages and may enhance overall the effects of therapy [45].

Etodolac, a selective cyclooxygenase-2 inhibitor has been shown to significantly reduce RAGE expression in dysplastic cells and carcinomas. Although the mechanism of inhibition remains unknown, it is possibly attributed to the reduction in AGE formation [46], which is usually induced by various inflammatory processes that can be inhibited by etodolac as part of its anti-inflammatory effect. Alternatively, etodolac may inhibit HMGB1 and its gene products [47,48,49], such as amphoterin which is an activator ligand of RAGE. Therefore, Etodolac’s chemopreventive effect is possibly related to RAGE downregulation in 4-NQO (4-Nitroquinoline1-oxide) induced carcinogenesis [50]. Further data advocating the hypothesis that RAGE-HMGB1 axis could be a potential drug target come from in vitro experiments where administration of the calcium channel blocker, nifedipine in a murine OSCC cell line (SCC7 cells) was shown to inhibit the RAGE-HMGB1 interaction in a dose-dependent manner [51]. Nifedipine, being an inhibitor of the L-type voltage gated calcium channels has been used to lower blood pressure and increase the supply of oxygen to the heart. In SCC7 cells, the interaction of RAGE-HMGB1 was shown to be associated with the migration of cancer cells and upon nifedipine’s administration this effect was reduced, suggesting a potential inhibitory role of nifedipine in cancer metastasis [51]. 

Additionally, Evodiamine (EVO), a natural alkaloid compound with anti-proliferative activity was tested in OSCC and was shown to downregulate the expression of both HMGB1 and RAGE in vitro and in vivo (Table 1). EVO administration in mice injected with HSC4 cells exhibited suppression of tumor growth, invasion and proliferation indicating that the anti-tumor effects of EVO may be mediated through inhibition of the HMGB1/RAGE signaling pathway [31].

Other natural compounds that may inhibit RAGE-HMGB1 interaction include glycyrrhizin (GL), a triterpenoid compound of licorice extract and Papaverine, a benzylisoquinoline alkaloid of the *Papaverine somniferum* plant [52,53]. GL has been shown to regulate cancer cell death, oxidative stress, and inflammation by modulating various signaling pathways, such as MAPK, phosphatase and tensin homolog/phosphatidylinositol 3-kinase/protein kinase B pathway (PTEN/PI-3K/PKB), and the mammalian target of rapamycin/signal transducer and activator of transcription 3 (mTOR/STA3) axis in several cancer cell types [52]. 

Moreover, papaverine, clinically used as a vasodilator has been shown to increase cyclic adenosine 3′, 5′-monophosphate (cAMP) levels and affect intracellular signaling pathways including the inflammatory HMGB1/RAGE interaction, suppressing the formation of pro-inflammatory cytokines [54], PI3K/Akt, mTOR and VEGF as well as mitochondrial metabolism [53]. Papaverine has been demonstrated to reduce cell proliferation of adenocarcinoma alveolar cancer cells, human hepatoma and OSCC cell lines and has been suggested as a potential anti-cancer and anti-inflammatory agent that needs to be further explored [53,54,55].

**Table 1:** Drugs/molecules used in experimental studies to attenuate HMGB1 effects in oral cancer.

Drugs	Molecule/Target	Action	Reference
NecroX-7	HMGB1	Decrease in the severity and incidence of ulceration in OM	[26]
JQ1	HMGB1	HMGB1 release	[42]
3-MA/YC-1	PI3K/HIF-1α/ BNIP3/Beclin1	Attenuation of autophagy	[29]
HMGB1 antibody	HMGB1	Macrophage recruitment	[45]
Etodolac	COX-2	Inhibition of HMGB1 through downregulation of RAGE	[47,48,49]
Evodiamine	HMGB1, RAGE	Tumor growth suppression	[31]
Nifedipine	RAGE-HMGB1 interaction	Reduction in cancer cell migration	[51]
Papaverine	RAGE-HMGB1 interaction	Inhibition of cancer cell proliferation	[55]

NecroX-7, tetrahydropyran-4-yl-[2-phenyl-5-(1,1-dioxo-thiomorpholin-4-yl) methyl-1Hindole-7-yl] amine; 3-MA, 3-Methyladenine; PI3K, phosphatidylinositol 3-kinase; HIF-1α, hypoxia-inducible factor 1-alpha; BNIP3, BCL2 interacting protein 3; COX-2, cyclooxygenase-2; RAGE, receptor for AGE.

## 5. Conclusions

Oral cancer belongs to the list of the most common cancers affecting millions of people around the globe, with 65% reaching the five-year survival but only 27% of advanced-stage patients [32]. Molecular characterization and treatment regimens are still in progress seeking further investigation and continuous research efforts.

However, accumulating research data revealed that the upregulation of HMGB1 in OSCC is involved in the development and pathology of oral cancer as well as in chemotherapy-associated oral mucositis. HMGB1, being a nuclear nonhistone protein and an extracellularly secreted cytokine can interact with EPHB4, NF-κB, TLR4 and MIA, to promote cancer cell proliferation, migration, metastasis, innate immune response, and tumor escape while also affecting autophagy. On the other hand, through its predominant ligand RAGE, it can activate intracellular signaling to enable cell proliferation, transmit pain signals, induce EMT pathway and metastasis, as well as bone invasiveness and angiogenesis leading to OSCC progression. The ability of HMGB1 to employ alternative ligands in order to mediate its activity makes it an attractive biomarker for OSCC progression and prognosis as well as a promising target for therapy. 

To this end, it is very important to characterize HMGB1 function in the OSCC context, for a better understanding of the dynamics of HMGB1 regulation. Pre-clinical studies should be designed to provide useful and accurate information of HMGB1 malignant potential, and ways of modulating OSCC aggressiveness to enable the development of novel diagnostics. 

Since several molecules and compounds have been already revealed (Table 1) to exert their influence in OSCC by targeting HMGB1, standardized and randomized validation research studies need to be performed to establish the specificity, sensitivity, and robustness of HMGB1 effects on tailored patients’ conditions and enhance the development of novel potential therapeutic agents targeting HMGB1.

## Data Availability

Not applicable.
